# LncRNA RNCR3 Promotes the Progression of HCC by Activating the Akt/GSK3*β* Signaling Pathway

**DOI:** 10.1155/2020/8367454

**Published:** 2020-09-29

**Authors:** Qi Xie, Tongfa Ju, Chunhua Zhou, Lulu Zhai

**Affiliations:** ^1^Department of General Surgery, Affiliated Hangzhou First People's Hospital, Zhejiang University School of Medicine, Hangzhou 310006, China; ^2^Department of General Surgery, Hospital of Wuhan University, Wuhan 430060, China

## Abstract

**Objective:**

To explore the potential biological roles of long noncoding RNA (lncRNA) RNCR3 in human hepatocellular carcinoma (HCC).

**Methods:**

First, the expression of RNCR3 was detected by qRT-PCR. Then, *in vitro* experiments were performed to investigate the effects of RNCR3 on the proliferation, cell cycle, migration, and invasion of HCC cells, while the effects of RNCR3 on HCC tumor growth and metastasis were investigated using *in vivo* experiments. Finally, western blot was used to study the activation of the Akt/GSK3*β* signaling pathway.

**Results:**

RNCR3 was highly expressed in both HCC tissues and cells, and the expression of RNCR3 was closely related to tumor size, tumor number, TNM stage, and overall survival time. *In vitro*, RNCR3 served as an oncogene to promote cell proliferation, migration, and invasion, and *in vivo*, RNCR3 promoted the growth and metastasis of HCC tumors. In terms of mechanism, RNCR3 induced the phosphorylation of Akt (thr308 and ser473) and GSK3*β* (ser9) but decreased the expression of GSK3*β*, which activated the Akt/GSK3*β* signaling pathway.

**Conclusion:**

The high expression of lncRNA RNCR3 in HCC can promote the proliferation, migration, invasion, growth, and metastasis of HCC by activating the NF-*κ*B signaling pathway.

## 1. Introduction

Hepatocellular carcinoma (HCC) accounts for nearly 80% of all primary liver cancers, ranking third among cancer-related deaths, and a large number of patients die from HCC every year globally [1, 2]. Surgical resection is still the main choice for patients with resectable HCC, but due to the rapid tumor progression and intrahepatic and extrahepatic metastasis, the postoperative tumor recurrence rate is still very high, which leads to a poor prognosis [3]. Therefore, preventing the progress and metastasis of hepatocellular carcinoma is the most important task at present, and exploring the molecular mechanism of the occurrence and development of liver cancer is of great significance for finding new therapeutic targets.

Long noncoding RNA (lncRNA) is a kind of noncoding RNAs with no protein-coding ability, and its length exceeds 200 nucleotides [4]. It has been reported that lncRNAs are involved in various cellular processes including cell proliferation, apoptosis, cell cycle, and invasion, and more and more evidence shows that the abnormal expression of lncRNAs that served as oncogenes or tumor suppressor genes is related to the occurrence and development of various cancers [5, 6]. In HCC, many lncRNAs play an important role in the occurrence and development of tumors, indicating that lncRNAs can be used as diagnostic markers and therapeutic targets for HCC. For example, the expression of LINC01554 in HCC is significantly downregulated, and its expression has a significant relationship with the tumor size, multiple lesions, TNM stage, tumor recurrence rate, and long-term survival rate of liver cancer patients [7]. It is also reported that LINC00467 can promote the proliferation and invasion of HCC cells through miR-509-3p/PDGFRA, inhibit cell apoptosis, and contribute to Axitinib resistance of hepatocellular carcinoma, which indicates that LINC00467 is a promising biological target for the treatment of liver cancer [8].

Retinal noncoding RNA3 (RNCR3, also known as LINC00599) is a lncRNA transcribed from intergenic regions of the genome and is highly conserved among mammals [9]. Many studies have shown that RNCR3 plays a critical regulatory role in cell proliferation, differentiation, and atherosclerosis [10, 11]. In human cancer, RNCR3 as an oncogene promotes the progression of malignant tumors such as colorectal cancer, glioma, and prostate cancer [12–14]; however, the biological function and molecular mechanism of RNCR3 in HCC have not been fully elucidated. Therefore, this study will investigate the expression, biological effects, and corresponding mechanisms of RNCR3 in HCC.

## 2. Materials and Methods

### 2.1. Tissue Samples and Ethics

Both HCC cancer tissues and para-cancerous tissues were from patients undergoing surgical treatment in our hospital, and all patients signed informed consent. Before the sample collection, this study was approved by the ethics committee of our hospital. The para-cancerous specimen was taken from the tissue 3 cm away from the edge of liver cancer, after which the collected tissue was immediately placed in a RNA long-term preservation solution (Solarbio, China) and stored at −80°C.

### 2.2. RNA Isolation, Reverse Transcription (RT), and Real-Time Quantitative Polymerase Chain Reaction (qRT-PCR)

Total RNA was extracted from all tissues and cells using TRIzol reagent (Thermo Fisher Scientific, USA), and the concentration of the total RNA was quantified using a NanoDrop 2000 spectrophotometer (Thermo Fisher Scientific). After that, the RNA was reverse transcribed into cDNA using a PrimeScript RT kit (TAKARA, Japan), and the PCR reaction was performed on PikoReal 96 real-time PCR system (Thermo Scientific) using SYBR Green qPCR Master Mix (TAKARA, Japan). GAPDH was used as an internal reference, and the relative expression level of RNCR3 was calculated using the 2^−ΔΔCt^ method. Primer sequences are shown in Table 1.

### 2.3. Cell Culture and Transfection

Five human HCC cell lines (SMMC-7721, HepG2, Huh-7, HL-7702, and Huh-6) and human normal liver cell lines (HL-7702) were purchased from Shanghai Fuheng Biotechnology Co., Ltd. All cells were cultured in a DMEM high-glucose medium (Hyclone, USA), supplemented with 10% fetal bovine serum (Gibco, USA), 100 U/mL penicillin and 100 mg/mL streptomycin (Sigma, USA), and incubated in a humid environment containing 5% CO_2_ at 37°C. RNCR3 overexpression plasmids or shRNAs were transfected into cells using Lipofectamine 2000 (Life Technologies, USA).

### 2.4. CCK8 Experiment

Cell proliferation ability was measured using the CCK8 kit (Dojindo, Japan). First of all, cells were seeded in 96-well plates at a density of 1 × 10^3^ cells/well and cultured for 24 to 72 hours. After that, 10 *μ*L of CCK8 solution was added to each well and incubated at 37 °C for an appropriate time, and then, the absorbance at 450 nm was measured with a spectrophotometer.

### 2.5. Cell Cycle Analysis

First, the cells were washed in PBS and fixed with 75% ethanol at 4 °C overnight. Then, the cells were stained with PI/RNase staining buffer (BD Biosciences, USA). Finally, the cells were analyzed by flow cytometry (BD Biosciences), and the proportion of G1, S, and G2 phase cells was compared.

### 2.6. Scratch Test

The scratch test was used to study the effect of RNCR3 on cell migration. First, the transfected cells were seeded in 12-well plates, to be grown to a density of 80–90%, and then, the same width of scratches was scratched with the tip of a sterile 10 *μ*L pipette. After that, the cells were washed with PBS solution to remove the crossed-out cells, and then, the cells were cultured with a serum-free medium for 24 hours. Images were taken using an optical microscope (Olympus, Japan) at 0 hours and 24 hours after scratching.

### 2.7. Transwell Experiment

The transwell experiment was used to determine the cell invasion ability. First, the transfected cells (5 × 10^4^ cells per well) were added to the upper chamber of the transwell chamber (Corning, USA) precoated with matrix gel (BD Biosciences) and cultured in a serum-free medium, and then the DMEM medium containing 10% FBS was added into the lower chamber. After incubating at 37°C for 24 hours, the upper chamber cells were removed with a cotton swab, and the cells that penetrated the membrane were fixed with methanol and stained with 0.1% crystal violet for 5 min. Finally, the stained cells were photographed using an optical microscope (Olympus) and then were counted.

### 2.8. Western Blot Experiment

Cells were lysed using RIPA buffer (Beyotime, China) to extract proteins. First, the protein samples were electrophoresed by SDS-PAGE, and the proteins were transferred to the PVDF membrane. Then, after incubating the membrane with the primary antibody, the secondary antibody was incubated. Finally, an ECL chemiluminescence kit (Beyotime) was used for imaging.

### 2.9. Tumor Formation Experiment in Nude Mice

Four-week-old BALB/c nude mice used in this study were purchased from Changzhou Cavens Experimental Animal Co., Ltd. The animal experiments in this study were approved by the Animal Ethics Committee of this hospital. First of all, 2 × 10^7^ HepG2 cells stably transfected with sh-NC or shRNCR3 were injected subcutaneously into nude mice. Then, the tumor volume was measured and calculated every 5 days using a digital caliper, and the formula was as follows: volume = (length × width ^2^)/2. After 20 days, the nude mice were sacrificed and the subcutaneous tumors were excised and implanted into new nude mouse livers. Five weeks later, the new nude mice were sacrificed, and lung tissue sections were taken and stained with HE to observe the tumor metastasis.

### 2.10. Statistical Analysis

All experiments were repeated at least three times, and the data were expressed as mean ± standard deviation. All analyses were performed using SPSS 20.0 software (SPSS, USA). Differences between groups were analyzed using *t*-test or one-way ANOVA, and the Kaplan–Meier method and Log-rank test were used for survival analysis. *P* < 0.05 was considered statistically significant.

## 3. Results

### 3.1. High Expression and Clinical Significance of RNCR3 in HCC

**Figure 1:**
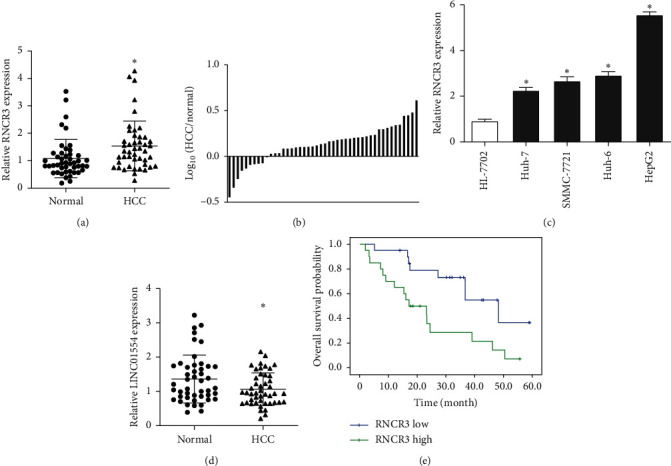
High expression of RNCR3 in HCC and its effects on prognosis. (a, b) Comparison of RNCR3 expression in HCC tissues and normal liver tissue adjacent to cancer. (c) Comparison of RNCR3 expression in HCC cell lines and human normal liver cell lines, *n* = 3. (d) Comparison of the expression of LINC01554 in HCC tissue and normal liver tissue adjacent to cancer. (e) Overall survival curve of patients with high and low RNCR3 expression. ^*∗*^*P* < 0.05.

First, 46 pairs of HCC tissues and normal liver tissues adjacent to cancer were used to detect the RNCR3 expression by qRT-PCR. The results showed that the expression of RNCR3 in HCC tissues was significantly higher than that in normal tissues (Figures 1(a) and 1(b)). Furthermore, the expression of RNCR3 in the HCC cell line was significantly higher than that of the human normal liver cell line (Figure 1(c)). These results indicated that RNCR3 was highly expressed in HCC. The expression of lncRNA LINC01554, which has been reported in HCC, was also tested as a positive control, and it was found that the LINC01554 expression was downregulated in HCC tissues (Figure 1(d)). To explore the clinical significance of RNCR3 expression, the expression of RNCR3 was divided into a high-expression group and a low-expression group according to the expression median and compared with clinical factors. It was found that the expression of RNCR3 was closely related to tumor size, tumor number, and TNM stage (Table 2). Through survival analysis, it was found that patients with the high expression of RNCR3 had a shorter overall survival time (Figure 1(e)).

### 3.2. RNCR3 Overexpression and Knockout Verification *In Vitro*

**Figure 2:**
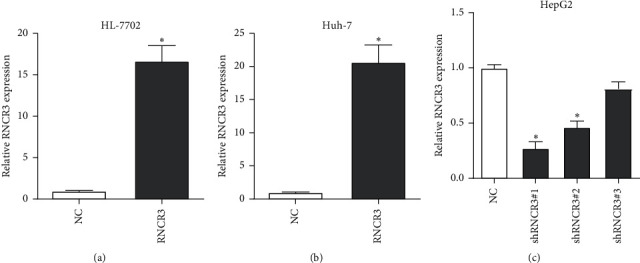
RNCR3 overexpression and knockout verification *in vitro*. (a–c) qRT-PCR experiment to detect the expression of RNCR3 after transfection of RNCR3 expression plasmid or shRNA. ^*∗*^*P* < 0.05, *n* = 3.

Due to the low expression of RNCR3 in HL-7702 cells and Huh-7 cells and high expression of RNCR3 in HepG2 cells, to study the biological function of RNCR3, the RNCR3 overexpression plasmid was transfected in HL-7702 cells and Huh-7 cells, while RNCR3 shRNAs were transfected in HepG2 cells, and the qRT-PCR test was used to detect for verification. The results suggest that RNCR3 can be successfully overexpressed in HL-7702 and Huh-7 cells (Figure 2(a) and 2(b)), and shRNCR3#1 had the best silencing efficiency (Figure 2(c)), so this shRNA was selected for subsequent experiments.

### 3.3. RNCR3 Promotes HL-7702 Cell and HCC Cell Proliferation *In Vitro*

**Figure 3:**
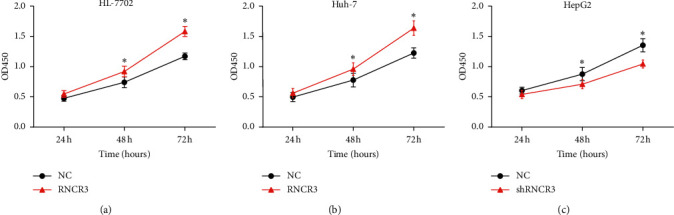
RNCR3 promotes HL-7702 cell and HCC cell proliferation *in vitro*. (a–c) CCK8 experiment was performed to detect cell proliferation. ^*∗*^*P* < 0.05, *n* = 3.

The overexpression plasmid was transfected in HL-7702 and Huh-7 cells, and RNCR3 shRNA was transfected in HepG2 cells. The CCK8 test was used to detect the changes in cell proliferation ability. The results showed that overexpression of RNCR3 can significantly increase the proliferation capacity of HL-7702 cells and Huh-7 cells (Figure 3(a) and 3(b), but silencing RNCR3 inhibited the proliferation of HepG2 cells (Figure 3(c)). This result suggested that RNCR3 can promote HCC cell proliferation *in vitro*.

### 3.4. RNCR3 Regulates HL-7702 Cell and HCC Cell Cycle *In Vitro*

**Figure 4:**
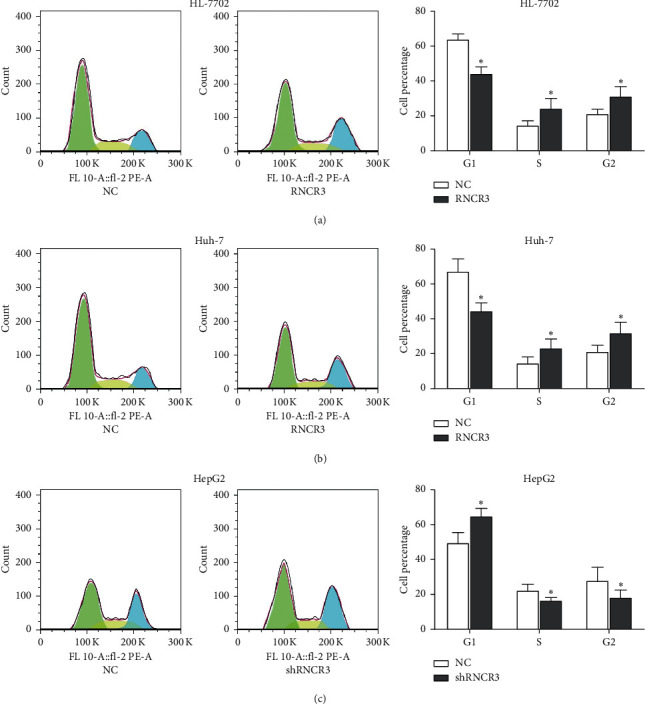
RNCR3 regulates the HL-7702 cell and HCC cell cycle *in vitro*. (a–c) Cell cycle experiment was performed to detect the percentage of cells in each stage. ^*∗*^*P* < 0.05, *n* = 3.

The RNCR3 overexpression plasmid was transfected in HL-7702 cells and Huh-7 cells, RNCR3 shRNAs were transfected in HepG2 cells, and flow cytometry was used to detect changes in the cell cycle. The results showed that overexpression of RNCR3 can significantly increase the percentage of HL-7702 cells and Huh-7 cells in S and G2 phases (Figure 4(a) and 4(b)), while silencing RNCR3 decreased the percentage of HepG2 cells in S and G2 phases (Figure 4(c)). This result indicated that RNCR3 can regulate the HCC cell cycle *in vitro*.

### 3.5. RNCR3 Promotes HL-7702 Cell and HCC Cell Migration *In Vitro*

**Figure 5:**
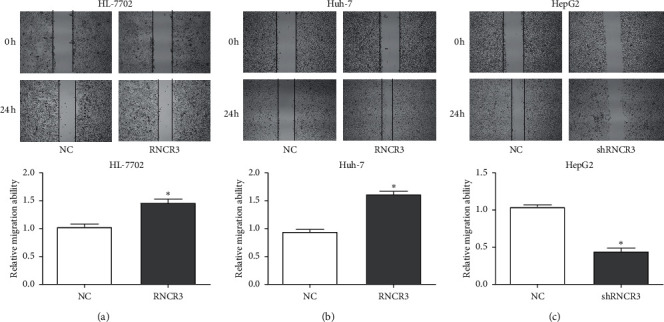
RNCR3 promotes HL-7702 cell and HCC cell migration *in vitro*. (a–c) The scratch test was used to evaluate the effect of RNCR3 on cell migration. ^*∗*^*P* < 0.05, *n* = 3.

The RNCR3 overexpression plasmid was transfected in HL-7702 cells and Huh-7 cells, RNCR3 shRNAs were transfected in HepG2 cells, and the changes in cell migration ability were investigated using scratch experiments. The results showed that overexpression of RNCR3 can promote the migration of HL-7702 cells and Huh-7 cells (Figure 5(a) and 5(b)), while silencing RNCR3 inhibited the migration of HepG2 cells (Figure 5(c)). This result suggested that RNCR3 can promote HCC migration *in vitro*.

### 3.6. RNCR3 Promotes HL-7702 Cell and HCC Cell Invasion *In Vitro*

**Figure 6:**
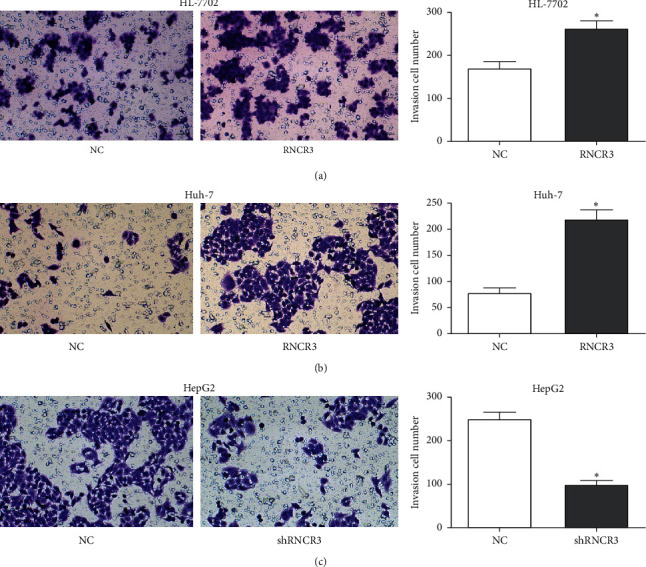
RNCR3 promotes HL-7702 cell and HCC cell invasion *in vitro*. (a–c) Transwell experiment was performed to evaluate the effect of RNCR3 on cell invasion. ^*∗*^*P* < 0.05, *n* = 3.

The RNCR3 overexpression plasmid was transfected in HL-7702 cells and Huh-7 cells, RNCR3 shRNAs were transfected in HepG2 cells, and the changes in cell migration ability were evaluated using the transwell experiment. The results showed that overexpression of RNCR3 can promote the invasion of HL-7702 cells and Huh-7 cells (Figure 6(a) and 6(b)), while silencing RNCR3 inhibited the invasion of HepG2 cells (Figure 6(c)). This result suggested that RNCR3 could promote the HCC invasion *in vitro*.

### 3.7. RNCR3 Promotes HCC Growth and Metastasis *In Vivo*

**Figure 7:**
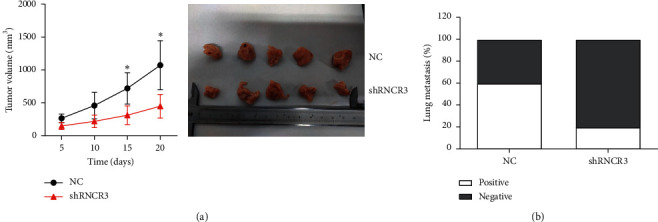
RNCR3 promotes HCC growth and metastasis *in vivo*. (a) Comparison of the growth rate and tumor size of subcutaneous tumors in nude mice. (b) Comparison of lung metastasis of liver cancer in nude mice. ^*∗*^*P* < 0.05, *n* = 5.

HepG2 cells stably transfected with sh-NC or shRNCR3 were injected subcutaneously into nude mice, and the growth rate of subcutaneous tumors was observed in nude mice. The results showed that the growth rate of the tumor was significantly slowed down after silencing RNCR3 (Figure 7(a)). Moreover, after silencing RNCR3, the lung metastasis ability of the tumor was significantly reduced (Figure 7(b)). This result indicated that RNCR3 can promote HCC growth and metastasis *in vivo*.

### 3.8. RNCR3 Can Activate the Akt/GSK3*β* Signaling Pathway

**Figure 8:**
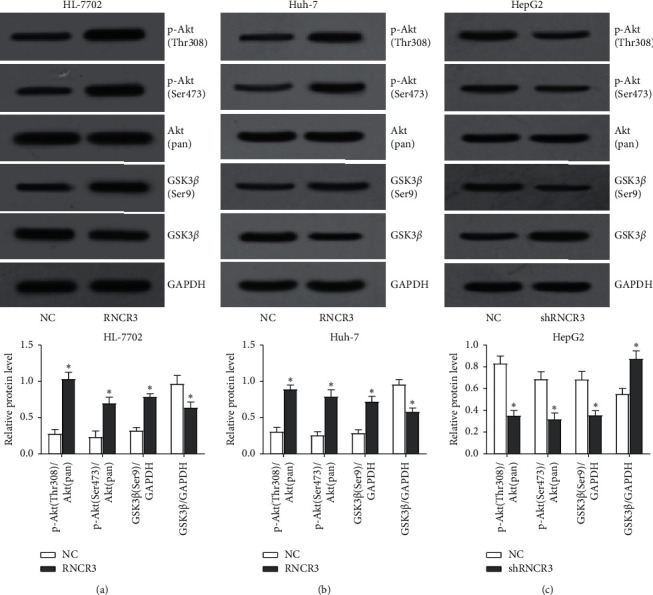
RNCR3 can activate the Akt/GSK3*β* signaling pathway. (a–c) Western blot experiments were used to detect protein expression changes in Akt/GSK3*β* signaling pathway. ^*∗*^*P* < 0.05, *n* = 3.

Studies have shown that RNCR3 can activate the Akt/GSK3*β* signaling pathway in gliomas to exert its cancer-promoting effect [13], so we verified the effect of RNCR3 on the Akt/GSK3*β* signaling pathway in HCC. The RNCR3 overexpression plasmid was transfected in HL-7702 cells and Huh-7 cells, RNCR3 shRNAs were transfected in HepG2 cells, and the protein expression changes of the Akt/GSK3*β* signaling pathway was measured by western blot. The results showed that overexpression of RNCR3 can promote the phosphorylation of Akt (Thr308 and Ser473) and GSK3*β* (Ser9) but reduce the expression of GSK3*β* (Figure 8(a) and 8(b)). However, silencing RNCR3 inhibited the phosphorylation of Akt (Thr308 and Ser473) and GSK3*β* (Ser9) but upregulated the expression of GSK3*β* (Figure 8(c)). This result suggested that RNCR3 can activate the Akt/GSK3*β* signaling pathway in HCC.

## 4. Discussion

In the past few years, the lncRNA has been a research hotspot, and its discovery has greatly changed our biological understanding of many complex diseases [15]. To date, HCC still has a poor prognosis and a high mortality rate, and its prevention and treatment have been troublesome for many researchers. Therefore, it is of great significance to explore lncRNAs in HCC.

This study focuses on the expression and role of lncRNA RNCR3 in HCC. Our results showed that RNCR3 was highly expressed in HCC tissues and cells, and the expression of RNCR3 was closely related to tumor size, tumor number, TNM stage, and overall survival time. It has been reported that the expression of RNCR3 in glioma tissues was increased compared with the corresponding adjacent normal tissues, and the increase in its expression was related to the progression of the tumor and the low survival rate of glioma patients [13]. Moreover, the expression of RNCR3 in prostate cancer was also increased, and its high expression was significantly associated with tumor progression and low survival rate of patients with prostate cancer [14]. These results indicate that RNCR3 can be used as a potential biomarker in clinical applications.

Our *in vitro* experiments showed that RNCR3 can be used as an oncogene to promote cell proliferation, migration, and invasion. Further *in vivo* experiments found that RNCR3 promotes HCC growth and metastasis. In many other tumors, RNCR3 also plays the role of an oncogene; for example, in colorectal cancer, RNCR3 can promote tumor cell proliferation, colony formation, and invasion and inhibit apoptosis [12]. In glioma, RNCR3 can increase the proliferation and invasion ability of glioma cells [13]. In this study, animal experiments were added to confirm that RNCR3 promotes HCC growth and metastasis *in vivo*; therefore, the biological function of RNCR3 in HCC was clarified both *in vitro* and *in vivo*.

In terms of mechanism, our results demonstrated that RNCR3 can promote the phosphorylation of Akt (Thr308 and Ser473) and GSK3*β* (Ser9) but reduce the expression of GSK3*β*, thereby activating the Akt/GSK3*β* signaling pathway. AKT, also known as protein kinase B, is a known oncogene whose abnormal overexpression or activation is found in many cancers and is associated with cancer cell proliferation, increased survival rate, invasion, and migration [16]. The activation of Akt depends on the phosphorylation of Thr308 and Ser473 and the activated Akt can inactivate GSK-3 by phosphorylating a single residue on GSK3*β* Ser9 [17]. GSK-3 is a versatile serine/threonine kinase that was originally considered to be a regulator of glycogen metabolism. It is now believed that GSK-3 plays various important functions in cell division, proliferation, differentiation, and adhesion [18]. GSK-3 has been shown to regulate cyclin D1 proteolysis and subcellular localization, thereby affecting cell cycle and cell proliferation [19]. Moreover, GSK-3 can also affect the expression of EMT-related genes, thereby regulating cell migration and invasion [20]. Therefore, we believe that RNCR3 can promote the proliferation, migration, invasion, growth, and metastasis of HCC cells by activating the Akt/GSK3*β* signaling pathway.

## 5. Conclusion

Our research shows that lncRNA RNCR3 is highly expressed in HCC, and RNA3 can promote the proliferation, migration, invasion, growth, and metastasis of HCC cells by activating the Akt/GSK3*β* signaling pathway. Therefore, RNCR3 is expected to become a biomarker and therapeutic target for HCC.

## Figures and Tables

**Table 1:** Primer sequences used in PCR reaction.

Gene	Forward primer (5′-3′)	Reverse primer (5′-3′)
RNCR3	CAACACCTTCCTCCGTGACTGTG	GCTGGCTCCTTCTTGTCCACATA
LINC01554	GGAGGTCGGTTGATGAGCAGT	GTCAAGCCTGTGTGTCATCGTT
GAPDH	TCGACAGTCAGCCGCATCTTCTTT	ACCAAATCCGTTGACTCCGACCTT

**Table 2:** Relationship between RNCR3 expression and clinical factors in HCC patients.

Factor	Total number	RNCR3 high	RNCR3 low	*P* value
Sex				
Male	41	21	20	
Female	5	2	3	1.0
Age (years)				
≤60	33	15	18	
>60	13	8	5	0.326
AFP (ng/mL)				
≤20	21	12	9	
>20	25	11	14	0.375
ALT (U/L)				
≤40	27	15	12	
>40	19	8	11	0.369
Cirrhosis				
No	6	2	4	
Yes	40	21	19	0.665
TNM stage				
I	34	14	20	
II-III	12	9	3	0.044
Tumor size (cm)				
≤5	33	13	20	
>5	13	10	3	0.022
Number of tumors				
1	35	14	21	
≥2	11	9	2	0.016
Microvascular invasion				
No	31	13	18	
Yes	15	10	5	0.116
HBV				
Negative	8	3	5	
Positive	38	20	18	0.744

The data were analyzed using the chi-square test or Fisher's exact test.

## Data Availability

All data generated or analyzed during this study are included in this published article.
